# Using Social Media Marketing to Improve Retention of Children in the Special Supplemental Nutrition Program for Women, Infants, and Children: Implementation Study

**DOI:** 10.2196/77172

**Published:** 2025-12-10

**Authors:** Eriko M Robinson, Carla J Sabugo, Carolina Crisafi, Susan M Gross, Yunhee Kang, Elisabet V Eppes, Marycatherine Augustyn, Karen Castellanos-Brown, David M Paige, Laura E Caulfield

**Affiliations:** 1 Florida Department of Health in Miami-Dade County, WIC and Nutrition Program, Miami, FL, United States; 2 Department of Population, Family and Reproductive Health, Johns Hopkins Bloomberg School of Public Health, 615 North Wolfe Street, Baltimore, MD, United States; 3 Department of International Health, Center for Human Nutrition, Johns Hopkins Bloomberg School of Public Health, 615 North Wolfe Street, Room W2041, Baltimore, MD, 21205, United States, 1 240-460-3420; 4 Supplemental Nutrition and Safety Program Research and Analysis Evaluation Branch, Food and Nutrition Service, USDA, Alexandria, VA, United States

**Keywords:** social media, infants, children, Special Supplemental Nutrition Program for Women, Infants, and Children, WIC, program evaluation

## Abstract

**Background:**

Many eligible infants and children do not participate in the Special Supplemental Nutrition Program for Women, Infants, and Children (WIC); coverage declines throughout the preschool period of eligibility. National and state-level social marketing campaigns promote the value of WIC and increase enrollment and participation. Local contextualization and targeting of materials may increase effectiveness, considering the diversity of families eligible for the program. However, there are few examples of such approaches and their impact.

**Objective:**

This study evaluated the impact on child retention of a locally contextualized and targeted social media marketing campaign directed to WIC-eligible families living in the minority-majority population of Miami-Dade County, Florida.

**Methods:**

The digital marketing campaign geographically targeted low-income families with young children with customized static image and video advertisements on Facebook and Instagram, and a bilingual Google Ads campaign. It was implemented in 2 of 15 clinics operated by the Miami-Dade WIC local agency from May 2020 through April 2021. A before and after evaluation used program administrative data to compare the outcomes for infants and children in 2 innovation clinics (n=6162) with 11 comparison clinics (n=41,074) during a baseline period (2019 calendar year) and the implementation period (n=5636 and n=38,241, respectively). Outcome measures included recertification (re-enrollment during a period), retention (active in the program at the end of a period), and participation (household continuous benefit issuance defined as 11 out of 12 mo). Impact was assessed following cluster-adjusted propensity score weighting and difference-in-difference modeling. Household continuous benefit issuance was estimated in households with only an infant or a child.

**Results:**

Overall, 1,994,170 people were exposed to the campaign advertisements; 16.68% engaged with an advertisement. There were 22,983 unique visits to the local program website, 69.6% of which were acquired directly from the campaign. Four of the 5 top-performing advertisements were locally tailored messages and in Spanish. The change in recertification over time was 5.2% points (95% CI 3.4%-7.1%), greater for those in the innovation group than those in the comparison group. For retention and continuous benefit issuance, the absolute difference in change was 5.5% points (95% CI 3.7%-7.3%), and 6.6% points (95% CI 3.5%-9.7%), respectively. Differences in change over time associated with the innovation were qualitatively stronger for infants than for children; the difference in change for recertification was 7.6% points (95% CI 5.1%-10.1%) for infants and 4.0% points (95% CI 2.2%-5.9%) for children.

**Conclusions:**

Engaging low-income families with young children through a locally contextualized targeted media marketing campaign can improve retention of children in WIC.

## Introduction

The Special Supplemental Nutrition Program for Women, Infants, and Children (WIC) was established by the US Department of Agriculture (USDA) to improve the health of low-income women and children by providing nutritious supplemental foods, education, breastfeeding support, and referrals to health and social service programs [1]. Among income-eligible individuals, participation in WIC is associated with improved birth outcomes, child nutrition status, and child health outcomes, all of which have impacts lasting into adulthood [2,3].

Despite the documented benefits, participation and retention in the WIC program has been a national concern, particularly for children. Nationally, in 2019, it was estimated that 83.8% of eligible infants participated in WIC, but participation declined after the age of 1 year to 56.6%, with declines to 42.8% and 38% to reach only 22% of eligible children at the age of 4 years [4]. Research has identified common barriers to program enrollment and retention, which include lack of knowledge of the program and of eligibility requirements, social stigma, language barriers, as well as issues in service quality [5-10]. To address service quality barriers, the USDA has launched initiatives to modernize WIC, and a national media campaign was introduced to promote enrollment and retention [11,12].

Beyond a national campaign, there is a need to design, implement, and evaluate locally contextualized marketing approaches to enhance WIC participation and retention considering the diversity of families eligible for WIC [4]. This project was conducted by the Miami-Dade WIC local agency, which serves Miami-Dade County, a large and ethnically diverse metropolitan county in Florida. According to the US census, in 2020, there were 2,701,767 residents, of whom 14.1% were living in poverty [13]. Overall, 69% of the Miami-Dade County population identifies as Hispanic or Latino, and 3.7% identify as Haitian or Kreyol. Approximately 55% are foreign-born, and of those, 43% do not hold US citizenship. Three-quarters of the Miami-Dade population speak a language other than English in their home. Building on the national campaign and the USDA-supported opportunity provided by the Hopkins Participant Research Innovation Laboratory for Enhancing WIC Services, we designed and tested an integrated media marketing campaign that digitally and geographically targeted WIC-eligible families with customized static image and video advertisements on Facebook and Instagram, as well as a bilingual Google Ads campaign. Based on consumer buying behavior theory [14], it was hypothesized that a digital media campaign aimed at driving users to the Miami-Dade WIC website and appointment call center and increasing engagement and brand awareness could have a positive impact on child retention by “converting” the passive WIC enrollee or family that does not participate in benefit issuance, is late for recertification, or is terminated from the program after failure to recertify. Here we report on the implementation of the integrated media marketing campaign and the evaluation of its impact on the child recertification, benefit issuance, and retention in WIC.

## Methods

### Setting: The Florida Department of Health in Miami-Dade WIC Program

The Miami-Dade WIC Program has 15 service locations throughout the county with a total caseload of more than 50,000. Most clients live in the 4 to 5 zip codes surrounding the physical location of each clinic. For this project, 2 innovation clinics were selected that shared clients from the same zip codes and were similar in sociodemographic make-up. Total enrollment in WIC for these 2 sites in February 2019 was approximately 10,250. A total of 9 zip codes represented >75% of the caseload for these 2 sites, and of these, 7 were specific to the innovation sites and had limited overlap with clients that received services at other Miami-Dade WIC sites. These 7 ZIP codes were selected as target zip codes. Of the 13 remaining clinics, 2 clinics served a substantial number of clients from the same target zip codes; to reduce the likelihood of client crossover, these clinics were removed from the study. Because only small numbers of clients at the remaining 11 WIC clinics resided in the targeted zip codes, these clinics formed the comparison group.

### Social Media Marketing Framework

The integrated digital marketing campaign was developed as an innovative tool to retain and recruit potentially eligible families with infants and children aged 1 to 3 years. The overall design process and campaign materials are described elsewhere [15], and key aspects of the approach are described in this section. The framework integrated different methods to reach the target audience across different social media platforms in a coordinated fashion over time. The target audience received the campaign via four methods: (1) behavioral targeting, (2) social media marketing, (3) search engine marketing, and (4) mobile geo-precise strategy. Behavioral targeting refers to the use of historical behavior to customize the presentation of advertisements. For example, previous browsing history and interaction on advertisements for “picky eating” toddler play, toys for children aged 1 year, and potty training meet the target profile for parents of children aged 1 to 3 years. Layering income and sociodemographic data with behavioral data helps to identify existing and potential WIC clients. Marketing through social media platforms such as YouTube, Facebook, and Instagram targets users and their social circles, and the opportunity for sharing and “likes” increases engagement and allows for connections with other digital users, substantially increasing the reach of the campaign. This project used paid search advertisements on Google; we tested and used unique keywords specific to the project target audience (caregivers and parents of children aged 1-3 years, cultural and Spanish language keywords, and keywords specific to the geographic community and location). Finally, mobile geotargeting involves the use of privacy-compliant location data from cell phones, iPads, or computer IP addresses to present advertising. Users who lived, worked, or moved throughout the geotargeted areas were presented with WIC retention advertisements if they met all other criteria (either via social media or search engine).

Changes were made to the Miami-Dade WIC website and content to increase visibility and improve rankings in search engine results. All website content in English was professionally translated to both Spanish and Creole, the primary languages spoken in Miami-Dade County. Priority was placed on ensuring ease of access to the website (a short domain was purchased in lieu of the long state URL) as well as ease of use while on the website (appropriate reading level, content placement, navigation, accessibility of downloads, and contact information).

### Media Content

The Miami-Dade WIC Program obtained the rights to use and curate digital media content from the existing national campaign and developed custom creative content for the local campaign. A local WIC Advisory Committee made up of a diverse group of staff was established to provide insights, guidance, and recommendations for selected campaign messaging and content, to ensure the digital media campaign would be culturally, visually, and linguistically sensitive and meet the unique needs of the Miami-Dade population. A guide was created to provide direction on language, relatable imagery, and messaging needs, and further defined culturally acceptable material specific to the Miami-Dade community. All selected social media campaign material was also formally vetted and approved by USDA prior to posting. The Google Ads campaign keywords were developed with input from the vendor, existing keywords from the national campaign, and the WIC Advisory Committee.

### Implementation Timeline

The social media campaign was implemented over a 1-year period, beginning in May 2020, in 4 batches, with each batch consisting of 6 static advertisement posts and 2 videos. Batches 1 and 3 presented custom creative digital content, whereas batches 2 and 4 presented customized digital content from the existing national campaign. Performance was reviewed after each batch and edits to the content and advertisement strategy were made accordingly. For example, after reviewing language performance in batch 1, all new posts included Spanish translation in the social media caption incorporating a Hispanic dialect specific to the Caribbean and Central American population served. The Google Ads campaign began in May 2020, and keyword performance was monitored monthly. Facebook and Instagram advertisements were launched in mid-June 2020. The marketing vendor used software, internet platforms, and algorithms to behaviorally target advertisements. Targeting was also accomplished by testing various objectives, based on the desired outcome or goal for an advertisement. The campaign also tested various calls to action on the advertisement posts, which encouraged users to take specific actions on a social media post. After clicking the ad, the user would either be redirected to the Miami-Dade WIC website *or* be prompted to call the Miami-Dade WIC call center (click to call). In addition, performance based on content language, social media placement, run schedules, and creatives was assessed for each batch. Strategies that performed better were implemented in the upcoming batch.

*The pandemic* began between March 2020 and April 2020, and all Miami-Dade WIC clinics physically closed due to the COVID-19 response. Select sites remained open for limited in-person needs such as benefit card issuance, direct distribution issuance, and services for clients without access to email or phones. All other services were shifted to a virtual model which allowed Miami-Dade WIC to meet capacity and the needs of 100% of scheduled clients during the closures. In October 2020, all Miami-Dade WIC clinics reopened for in-person services as a complement to virtual services which continued to be an option for clients. The USDA federal waivers for COVID-19 remained in place through the end of the national emergency declaration (May 2023).

### Evaluation Design and Methods

The evaluation was designed to assess the performance of the innovative tool, and to assess the impact of the tool on relevant child outcomes in WIC.

### Process Evaluation

Marketing vendor data were used to assess the campaign’s reach, engagement, and conversion as key performance indicators (KPIs) [16]. Reach was defined as the number of unique users the advertisement is exposed to, and impression was defined by the number of views. Engagement occurs when a user likes, saves, shares, or comments on the post, and this is measured as a rate (engagements over impressions). When a user responded to a call-to-action and clicked on an advertisement either on social media or in the Google search engine, they were redirected either to the local agency website or the Miami-Dade WIC call center (depending on the predetermined media strategy). Total clicks, the click-through rate (CTR), and the share of website traffic attributable to the campaign measure conversion.

The original process evaluation plan also included in-clinic exit surveys to evaluate direct WIC client engagement with the campaign. When WIC services transitioned to a virtual service model due to the pandemic, the client engagement survey was necessarily moved to a virtual client-facing survey platform. The survey was administered in Spanish, Creole, or English, and clients were asked about where they had heard about WIC and whether they were familiar with Miami-Dade WIC social media pages. They were also asked if they had seen WIC advertisements on their cell phones or computers in the last month, and if so, they were asked to recall the platforms where they saw them (social media and Google). A competition was used to motivate staff to promote the survey during virtual certification appointments (via email link), but no client incentive was offered. Because the overall participant response rate was <5%, survey results are not presented here.

### Impact Evaluation

The impact evaluation focused on whether the integrated marketing tool increased the retention of enrolled infants and children aged 1 to 3 years. Program administrative data were obtained on cohorts of child WIC participants in innovation and comparison sites during a baseline period (prior to the innovation) and during the period when the innovation was implemented. This design, as previously described in Chancay et al [17], allowed for the evaluation of differences in outcomes between a baseline period and the implementation period, adjusting for changes over time common to both groups and group differences that do not vary over time. The baseline period was the 2019 calendar year (designated T1) and the implementation period was May 2020 through April 2021 (T2). Participant characteristics were also obtained to understand group differences and changes over time, and for use as covariates during analyses and included child age, sex, race and ethnicity, household size, number of children in WIC, primary language other than English, need for translation services, participation in other services (Medicaid, Supplemental Nutrition Assistance Program [SNAP], and Temporary Assistance for Needy Families).

Three outcomes were examined: recertification, retention, and benefit issuance. Data were obtained on WIC visits, recertification, and benefit issuance, for all infants (<12 mo) and children aged 12 to 47 months who were active in WIC in the innovation and comparison groups at the beginning of each period. Over a year-long period, each participant would have multiple visits, and benefits would be issued to them by WIC. In addition, the certification period for each child would end, and they would need to be recertified to continue in the WIC program. Through this approach, we could assess WIC recertification as an indicator of retention in cohorts of children and evaluate changes in the outcomes over time for the innovation group as compared to changes for the comparison group. To prevent censoring of outcomes for children whose certification period ended during months 11 or 12 of a period, data were obtained for an additional 2 months (that is, 14 mo of data were collected for each period), to identify those children who did recertify in a timely fashion, but after the end of the period. The WIC State agency also identified the participation status of each child at the end of a 12-month period as active, moved to another agency, inactive, or terminated; this status variable was used to define retention. Household WIC participation was also evaluated by continuous benefit issuance, defined as 11 to 12 months of benefit issuance over each 12-month period.

### Statistical Analysis

Descriptive analyses were conducted to describe the digital marketing campaign KPIs and to assess the comparability of participants in the innovation and the comparison groups within each period. Differences in performance and in participant characteristics were evaluated using 1- or 2-tailed *t* tests or chi-square tests as appropriate. We documented characteristics with a significant percentage of missing values (>10%), which might limit their usefulness during analysis. No values were imputed because we deemed it not necessary.

A difference-in-difference (DID) approach was used to assess program impact, which involved estimation of the changes over time in each outcome in the innovation versus the comparison group. Analyses were conducted for the overall sample as well as separately for infants and children. Propensity score weighting (PSW) was used to support adjustment for differences in participant characteristics between the innovation and comparison groups at each period (T1 and T2) as well as differences across the 2 periods. Weights were estimated using multinomial logistic regression in which observations were weighted as compared to the characteristics of those in the innovation group during T1 [17]. To evaluate the balance in participant characteristics achieved through weighting, we compared the absolute standardized differences (ASD) for the means of each participant characteristic before and after the weighting. This involved comparing the balance achieved through weighting for the innovation group over time (at T1 and T2), the innovation group at T1 and the comparison group at T1, and the innovation group at T1 with the comparison group at T2. These steps were repeated with samples restricted to infants and children. Final adjusted models for recertification and retention were also adjusted for clustering of households within clinics and of children within households. To fully present the results, we provide the crude and regression-adjusted means (with 95% CIs) for each outcome and the impact estimate as percentage points from each of two DID models: (1) DID (crude, unweighted) and (2) PSW-DID (adjusted) [18,19]. To evaluate household continuous benefit issuance, we restricted analyses to households with only 1 child WIC participant. This allowed us to consider results overall, for households with an infant, and for those with a child. In these analyses, adjustment was also made for clustering of households within clinics. The PSW step focused on household or caregiver characteristics.

### Ethical Considerations

The study design and methods were evaluated and determined to be public health practice and not research by the Florida State Department of Health Ethics and Human Research Protection Program. Deidentified administrative data were sent to Hopkins Participant Research Innovation Laboratory for Enhancing WIC Services for the impact evaluation analyses, which was deemed not to be research by the institutional review board at the Johns Hopkins Bloomberg School of Public Health.

## Results

### Process Evaluation

A total of 1,994,170 people were exposed to campaign advertisements, and 332,580 (16.68%) engaged with an advertisement (Google Analytics). There were 22,983 unique visits to the local WIC website, 15,996 (69.60%) of which were acquired directly from the campaign and represent the conversion rate. According to Google Analytics, the Google Ads campaign drove the highest number of users to the WIC website (13,149/15,996, 82.20%) followed by Facebook (2367/15,996, 14.80%). The mean number of impressions per month for the implementation period was 2330 (SD 209), and the average CTR was 20.95% (SD 1.6%). The top 5 performing key words (in terms of clicks to the website) in the Google Ads campaign were (1) “food stamps” (1243/8235, CTR=15%), (2) “WIC program” (909/2472, CTR=37%), (3) “wic” (713/2357, CTR=30%), (4) “comida gratis” (free food; 366/2324, CTR=16%), and (5) “EBT” (710/5283, CTR=13%).

Across all campaigns (Table 1), a greater share of impressions, views, and clicks were attributable to Facebook as compared to other social media platforms (Instagram, YouTube, and Audience Network 3rd party advertisements). Among the subset of advertisements run to direct traffic to the website or with a click to call or action, Facebook campaigns yielded a significantly higher overall CTR of 0.39% (4194/108,422) compared to 0.19% (1670/872,854) for all other platforms (*P*<.001). The average CTR for Facebook did not differ from the other platforms (*P*=.06).

**Table 1.** Overall performance of social media platforms during the campaign targeted to Special Supplemental Nutrition Program for Women, Infants, and Children (WIC)-eligible families in Miami-Dade, Florida, May 2020-April 2021. Results are presented by share of impressions, clicks and views, and average click-through rate.

Platform	Impressions (n=1,959,276)	Clicks (n=5864)	Engagement or views (n=319,863)	Average CTR^a^^b^ (SD)
Facebook, n (%)	1,086,422 (55.45)	4194 (71.52)	130,171 (40.70)	0.51 (0.46)^c^
Instagram, n (%)	421,156 (21.50)	933 (15.91)	10,615 (3.32)	0.39 (0.22)
YouTube, n (%)	450,370 (22.99)	734 (12.52)	177,739 (55.56)	0.17 (0.05)
Audience Network, n (%)	1328 (0.07)	3 (0.05)	1338 (0.42)	0.23^d^

a CTR: click-through rate. This is the average CTR (%) for advertisements running in traffic objective and click-to-call action on each platform.

b The average CTR for all campaigns is 0.47 (SD 0.41).

c Difference from other platforms (*P*=.06).

d This is the CTR and not average CTR.

Custom creative advertisements performed significantly better than existing national campaign advertisements (CTR of 0.38% vs 0.23% CTR; *P*<.001). Eligibility-themed content performed best with an average CTR of 0.49%, which was significantly greater than the other 3 themes (0.22%‐0.27% CTR; Table 2). Four of the 5 top-performing advertisements ran under the click-to-call or traffic objective, were custom creative type, and ran in the Spanish language. All the top-performing advertisements ran on Facebook (Table 3).

**Table 2.** Social media posts by theme, total clicks, and average click-through rate (CTR) as part of a social media campaign targeted to Special Supplemental Nutrition Program for Women, Infants, and Children (WIC)-eligible families in Miami-Dade, Florida, May 2020-April 2021.

Theme	Posts, n (%)^a^	Total clicks, n (%)	Average CTR (SD)
WIC added-value	46 (37.1)	2556 (43.59)	0.25 (0.20)
COVID-19	4 (3.2)	112 (1.91)	0.22 (0.12)
WIC eligibility	16 (12.9)	2027 (34.57)	0.49 (0.56)^b^
WIC misconceptions	18 (14.5)	1169 (19.93)	0.27 (0.21)

a Count includes individual advertisements run on multiple platforms and objectives as separate “posts.”

b Different from average CTR from other posts (*P*=.003).

**Table 3.** Top performing ads on Facebook during the campaign targeted to Special Supplemental Nutrition Program for Women, Infants, and Children (WIC)-eligible families in Miami-Dade, Florida, May 2020-April 2021. Performance is evaluated by click-through rate (CTR).

Advertisement title	Batch	Objective	Type	Theme	Language	Clicks/impressions	CTR (%)
Medicaid or SNAP^a^	3	Click to call	CC^b^	Eligibility	Spanish	786/44,454	1.77
Medicaid or SNAP—RERUN	4	Click to call	CC	Eligibility	Spanish	329/21,064	1.56
Welcome to WIC Video	3	Engagement	US^c^	Eligibility	English	340/23,995	1.42
Missing Meals	1	Traffic	CC	Added value	Spanish	168/15,566	1.08
Stay on WIC until your child is 5	1	Traffic	CC	Misconception	Spanish	217/26,056	0.83

a SNAP: Supplemental Nutrition Assistance Program

b CC: custom creative.

c US refers to the national campaign.

### Impact Evaluation

Shown in Table 4 are characteristics of the WIC participants in the innovation and comparison clinics during the baseline period (T1) and implementation period (T2). There were 3 potentially important differences between the innovation and comparison groups in Miami-Dade WIC. The innovation clinics at both T1 and T2 had fewer Black or African American participants (430/6162, 6.98% at T1 and 350/5636, 6.21% at T2) than the comparison clinics (12,793/41,074, 31.15% at T1 and 11,736/38,241, 30.69%), a greater number of White participants (5903/6162, 95.80% at T1 and 5411/5636, 95.97% at T2) than the comparison clinics (28,885/41074, 70.32% at T1 and 27,091/38,241, 70.84% at T2), and a greater number of Hispanic participants (5941/6162, 96.41% at T1 and 5496/5636, 96.10% at T2 in innovation clinics vs 28,625/41,074, 69.69% at T1 and 69.87 at T2 (26,719/38,241) in comparison clinics). There was a higher proportion of participants whose household language was something other than English (5031/6162, 81.64% at T1 and 4419/5636, 78.41% at T2 in the innovation clinics vs 20,823/41,074, 50.70% at T1 and 19,026/38,241, 49.75% at T2 in the comparison clinics). The only notable difference in participant characteristics within each group over time was a decline in SNAP participation. At T1, 49.60% (20,374/41,074) of the comparison group and 57.43% (3539/6162) of the innovation group participated in SNAP, but at T2, 37.95% (14,513/38,241) of the comparison group and 44.77% (2523/5636) of the innovation group were SNAP participants.

**Table 4.** Demographic characteristics of children 0‐3 at Miami-Dade Special Supplemental Nutrition Program for Women, Infants, and Children (WIC) comparison and innovation clinics at baseline period (T1) and implementation period (T2).

Characteristics		T1			T2		
		Comparison (n=41,074), n (%)	Innovation (n=6162), n (%)	*P* value^a^	Comparison (n=38,241), n (%)	Innovation (n=5636), n (%)	*P* value
Participant category				<.001			<.001
IBE^b^		1491 (3.63)	256 (4.15)		1401 (3.66)	229 (4.06)	
IBP^c^		5750 (14.00)	1048 (17.01)		5588 (14.61)	1121 (19.89)	
IFF^d^		7256 (17.67)	771 (12.51)		6624 (17.32)	624 (11.07)	
C1^e^		11,055 (26.92)	1643 (26.66)		10,162 (26.57)	1503 (26.67)	
C2^f^		8367 (20.37)	1388 (22.53)		7833 (20.48)	1172 (20.80)	
C3^g^		7155 (17.42)	1056 (17.14)		6633 (17.35)	987 (17.51)	
WIC participants				<.001			<.001
1		20,873 (50.82)	3327 (53.99)		20,576 (53.81)	3325 (59.00)	
2		6579 (16.02)	798 (12.95)		6673 (17.45)	964 (17.10)	
≥3		658 (1.60)	68 (1.10)		693 (1.81)	58 (1.03)	
Missing		12,964 (31.56)	1969 (31.95)		10,299 (26.93)	1289 (22.87)	
Race^h^							
American Indian or Alaska Native		58 (0.14)	14 (0.23)	.107	76 (0.20)	9 (0.16)	.534
Asian		383 (0.93)	32 (0.52)	.001	323 (0.85)	26 (0.46)	.002
Black or African American		12,793 (31.15)	430 (6.98)	<.001	11,736 (30.69)	350 (6.21)	<.001
Hispanic		28,625 (69.69)	5941 (96.41)	<.001	26,719 (69.87)	5416 (96.10)	<.001
Native Hawaiian or Other Pacific Islander		84 (0.20)	2 (0.03)	.003	82 (0.21)	0 (0.00)	.001
White		28,885 (70.32)	5903 (95.80)	<.001	27,091 (70.84)	5409 (95.97)	<.001
Enrolled							
TANF^ii^		569 (1.39)	63 (1.02)	.021	423 (1.11)	47 (0.83)	.064
SNAP^j^		20,374 (49.60)	3539 (57.4)	<.001	14,513 (38.0)	2523 (44.77)	<.001
Medicaid		38,362 (93.40)	5919 (96.1)	<.001	35,050 (91.7)	5296 (93.97)	<.001
Primary language other than English		20,823 (50.70)	5031 (81.7)	<.001	19,026 (49.8)	4419 (78.41)	<.001
Ever breastfed				<.001			<.001
Yes		32,042 (78.01)	5074 (82.34)		32,617 (85.3)	4918 (87.26)	
No		5077 (12.36)	596 (9.7)		4730 (12.4)	551 (9.78)	
Missing		3955 (9.64)	492 (7.98)		894 (2.34)	167 (2.96)	
Household size				<.001			<.001
0‐4		27,778 (67.63)	4441 (72.07)		26,505 (69.31)	4084 (72.46)	
≥5		12,982 (31.61)	1694 (27.49)		11,422 (29.87)	1511 (26.81)	
Missing		314 (0.76)	27 (0.44)		314 (0.82)	41 (0.73)	

a Difference between groups within a time period.

b IBE: infant, exclusive breastfeeding.

c IBP: infant, partial breastfeeding.

d IFF: infant, formula feeding.

e C1: child category 1 (aged 1 year).

f C2: child category 2.

g C3: child category 3.

h Participants can respond to more than 1 category, so the total percentage may be >100.

i TANF: Temporary Assistance for Needy Families.

j SNAP: Supplemental Nutrition Assistance Program.

PSW was successful in balancing differences between innovation and comparison groups and differences over time (Table S1 in Multimedia Appendix 1). After PSW, the ASDs were all reduced below 0.10, and the 3 mean ASDs were reduced to 0.01. This was also true for the analyses at the household level to evaluate continuous benefit issuance.

As shown in Table 5, the integrated media marketing campaign was associated with greater recertification and retention of children in WIC. Overall, the change in both recertification and retention was 5.2% to 5.5% points significantly greater among those exposed to the campaign than those in the comparison group, with significant differences in the change for these outcomes of 7.6% to 7.8% points among infants, and 4% to 4.4% points among children aged 1 to 3 years.

**Table 5.** Recertification and retention of infants and children in the innovation and comparison groups in the baseline and innovation periods using crude and adjusted models overall and for infants and children, and difference in difference (DID) estimate of innovation effectiveness.

Outcome	Baseline (T1)^a^		Implementation (T2)		DID
	Comparison	Innovation	Comparison	Innovation	
Overall					
Recertification (%)					
Observed^b^, n (%)	25,405 (63.23)	3826 (62.98)	26,793 (70.99)	4200 (75.39)	—^c^
Crude^d^, cluster adjusted^e^	63.9 (62.6, 65.2)	63.9 (60.6, 67.1)	64.7 (63.4, 66.0)	69.3 (66.0, 72.6)	4.6^f^ (3.0, 6.2)
PSW^d^, cluster adjusted	66.2 (65.2, 67.2)	62.9 (62.2, 63.6)	73.5 (71.7, 75.4)	75.4 (75.3, 75.5)	5.2^f^ (3.4, 7.1)
Retention (%)					
Observed, n (%)	24,822 (61.78)	3721 (61.25)	26,903 (71.29)	4203 (75.44)	—
Crude, cluster adjusted	62.6 (61.4, 63.9)	62.3 (59.1, 65.5)	64.6 (63.3, 65.8)	68.9 (65.7, 72.1)	4.6^f^ (3.0, 6.2)
PSW, cluster adjusted	64.9 (63.7, 66.2)	61.2 (61.0, 61.4)	73.8 (72.1, 75.5)	75.5 (75.5, 75.6)	5.5^f^ (3.7, 7.3)
Infants					
Recertification (%)					
Observed, n (%)	9579 (67.84)	1413 (69.13)	10,016 (74.73)	1557 (80.13)	—
Crude, cluster adjusted	67.8 (66.2, 69.3)	68.8 (64.8, 72.8)	73.3 (71.7, 74.9)	78.6 (74.6, 82.6)	4.3^h^ (1.3, 7.2)
PSW, cluster adjusted	73.9 (72.4, 75.4)	68.2 (64.8, 71.7)	78.2 (75.5, 80.9)	80.1 (75.8, 84.3)	7.6^f^ (5.1, 10.1)
Retention (%)					
Observed, n (%)	9283 (65.74)	1369 (66.98)	10,027 (74.81)	1552 (79.88)	—
Crude, cluster adjusted	65.8 (64.4, 67.3)	66.8 (63.0, 70.5)	73.4 (71.9, 74.9)	78.5 (74.7, 82.2)	4.1^i^ (1.2, 7.1)
PSW, cluster adjusted	72.2 (70.4, 74.0)	66.2 (63.5, 68.9)	78.2 (75.5, 80.8)	80.0 (75.8, 84.2)	7.8^i^ (5.1, 10.6)
Children					
Recertification (%)					
Observed, n (%)	15,826 (60.73)	2413 (59.84)	16,777 (68.94)	2643 (72.85)	—
Crude, cluster adjusted	60.9 (59.4, 62.4)	60.4 (56.6, 64.2)	63.5 (62.0, 65.0)	67.5 (63.7, 71.2)	4.4^f^ (2.4, 6.5)
PSW, cluster adjusted	62.5 (61.4, 63.6)	60.0 (59.2, 60.7)	71.5 (69.3, 73.7)	73.0 (72.0, 73.9)	4.0^f^ (2.2, 5.9)
Retention (%)					
Observed, n (%)	15,539 (59.63)	2352 (58.35)	16,876 (69.34)	2651 (73.07)	—
Crude, cluster adjusted	59.9 (58.4, 61.3)	59.1 (55.4, 62.8)	63.6 (62.1, 65.1)	67.3 (63.6, 71.1)	4.6^f^ (2.5, 6.6)
PSW, cluster adjusted	61.5 (60.3, 62.8)	58.5 (57.2, 59.7)	71.9 (69.8, 73.9)	73.2 (72.4, 74.1)	4.4^f^ (2.6, 6.2)

a Sample sizes for overall analyses are 40,181 and 6075 for comparison and innovation, respectively, at T1 and 37,740 and 5571, respectively, at T2. For infants, the corresponding sample sizes are 14,121 and 2044 at T1 and 10,016 and 1557 at T2, and for children, they are 26,060 and 4031 at T1 and 24,337 and 3,628 at T2.

b Observed values do not have applicable DID results.

c —: not applicable.

d Models were adjusted for clustering of children within households and households within clinics.

e The observed values are crude percentages. The crude and PSW are predicted values from regression models and their 95% confidence intervals.

f *P*<.001.

g PSW: propensity score weighting.

h *P*=.004.

i *P*=.006.

Among 67,198 households with only 1 child and using the PSW-DID adjusted model, the campaign was associated with 6.6% points greater change in continuous benefit issuance overall, with 7% points greater for households with an infant, and 5.9% points greater issuance for those with a child (Table 6).

**Table 6.** Continuous benefit issuance for households with only 1 child in the innovation and comparison groups in the baseline and innovation periods using crude and adjusted models overall, and for infants and children, and difference-in-difference (DID) estimate of innovation effectiveness.

Outcome	Baseline (T1)		Implementation (T2)		DID
	Comparison	Innovation	Comparison	Innovation	
Overall^a^					
Continuous benefit					
Issuance (%)					
Observed, n (%)^b^	14,675 (48.98)	2396 (50.22)	16,873 (59.98)	2882 (66.53)	—^c^
Crude, cluster adjusted^d^	48.9 (47.0, 51.0)	50.2 (49.2, 51.2)	60.0 (56.7, 63.2)	66.5 (66.2, 66.8)	5.3^e^ (2.6, 8.0)
PSW^f^, cluster adjusted	52.9 (51.8, 54.0)	50.2 (49.4, 51.0)	62.6 (59.3, 66.0)	66.6 (66.3, 67.0)	6.6^g^ (3.5, 9.7)
Infants^h^					
Continuous benefit					
Issuance (%)					
Observed, n (%)	5602 (51.19)	921 (56.16)	6530 (63.17)	1108 (71.76)	—
Crude, cluster adjusted	51.2 (49.3, 53.1)	56.2 (52.9, 59.4)	63.2 (60.4, 65.9)	71.8 (67.5, 76.0)	3.6^i^ (1.4, 5.9)
PSW, cluster adjusted	57.5 (56.1, 58.9)	56.2 (52.8, 59.6)	64.7 (61.8, 67.5)	70.3 (66.6, 74.2)	7.0^g^ (4.3, 9.7)
Children^j^					
Continuous benefit					
Issuance (%)					
Observed, n (%)	9073 (47.70)	1475 (47.11)	10,343 (58.12)	1744 (62.55)	—
Crude, cluster adjusted	47.7 (45.4, 50.0)	47.1 (43.8, 50.4)	58.1 (54.2, 62.0)	63.6 (61.5, 65.8)	6.1^e^ (3.1, 9.2)
PSW, cluster adjusted	50.5 (49.1, 51.9)	47.1 (44.0, 50.2)	61.1 (57.0, 65.1)	63.5 (61.9, 65.2)	5.9^k^ (2.4, 9.4)

a Overall household sample sizes at T1 for innovation and comparison are 4771 and 29,963, respectively, and for T2 are 4332 and 28,132, respectively.

b Observed values do not have applicable DID results.

c Not applicable.

d Models were adjusted for clustering of households within clinics.

e *P*=.001.

f PSW: propensity score weighting.

g *P*<.001.

h For households with 1 infant only, sample sizes at T1 for innovation and comparison are 1640 and 10,944 respectively, and for T2 are 1544 and 10,337 respectively.

i *P*=.004.

j For households with 1 child only, sample sizes at T1 for innovation and comparison are 3131 and 19,019 respectively, and for T2 are 2788 and 17,795 respectively.

k *P*=.003.

## Discussion

### Principal Results

To our knowledge, this is the first report of the impact of a multicomponent targeted social media campaign implemented by a local WIC agency to increase retention and participation of children in WIC. The digital marketing campaign reached a large, targeted audience, and the advertisements tailored to the local community had a higher engagement and drove a higher percentage of those who viewed it to the local agency site than the content created for a national audience. These findings add to the literature on the value of locally tailored messaging [20,21]. The results indicate that the Miami-Dade WIC social marketing campaign was associated with positive changes in recertification, retention, and continuous benefit issuance. In the adjusted analyses, overall recertification was 5.2% higher, with impacts on infants and children of 4.0% to 7.6%, and the impacts on retention were of similar magnitude. Household WIC participation as measured by continuous benefit issuance was 6.6% higher for the group exposed to the campaign than the comparison group. Together, these results indicate that targeted messaging tailored to the local community can successfully direct users to designated websites with trusted information or services and significantly impact continued participation in a program supporting maternal and child health.

There are few studies in the literature of media campaigns related to maternal and child health or related programs presenting results on digital KPIs of reach, engagement, or CTR. In an observational study of a media campaign to direct Canadian pregnant women to guidance on gestational weight gain [22], top keywords for clicking had CTR of 8% to 18%, while other top keywords with fewer clicks showed higher CTR of 18% to 25%. We also observed this pattern of CTR, but did also identify keywords with high numbers of clicks and a high CTR. We found the CTR for our top advertisements between 0.8% to 1.8%, with higher CTR for engagement and CTA objectives; these rates are comparable to those reported by Graham et al [22] among Canadian women (0.3%‐2.6%). Our conversion rate of 69.6% is lower but comparable to their reported conversion rate of 78.9%.

### Limitations

There are limitations to both the campaign and the evaluation. First, the social media campaign was targeted toward the innovation clinic geographic areas and WIC participant demographic characteristics, and therefore participant exposure to the innovation cannot be measured directly. We intended to use an in-person client survey to test recognition of campaign components, but this was not feasible with the transition to virtual service provision, and the low response rate limited its usefulness. In terms of the campaign itself, due to the strict 1-year timeline and the required content deliverables, the social media campaign was forced to test several strategies at one time (language testing, objective testing, and audience targeting), which diluted the accuracy of the results. Thoughtful testing of individual strategies and inclusion of WIC participants in that process would have provided valuable insights for future campaigns. The campaign was implemented in quarters, and analyses of performance metrics suggested stronger performance during some quarters than others. On the basis of the overall evaluation approach using administrative data to estimate annual rates, we could not assess or compare the impact by quarter. The focus of the evaluation was on retention of current WIC participants and was not designed to evaluate impact on enrollment in WIC. Finally, with only 1 year of preinnovation data, we are unable to evaluate the parallel trends assumption for the evaluation analyses, and despite our PSW adjustment, the possibility of unmeasured differences remains, thereby affecting our results. Related to this, we have no way of knowing whether or how the COVID-19 pandemic impacted the results.

### Conclusions

The study provides novel evidence that a locally tailored targeted social media outreach to WIC-participating families led to absolute improvements on the order of 4% to 7% in household continuous benefit issuance, rates of recertification, and the retention of children in WIC. Although the effect sizes are generally small, we would argue that these differences are important programmatically, given the importance of improving the retention of children in WIC, and findings from the published literature [23]. We provide initial estimates for planning future social media approaches to further test effectiveness on these outcomes. Future research should also evaluate these approaches to increase WIC coverage through new or re-enrollment in WIC. It would also be important to evaluate the impact on outcomes when implemented as a WIC State agency-level strategy.

## Supplementary material

10.2196/77172 Multimedia Appendix 1 Absolute standardized differences in characteristics.

## Abbreviations

ASD: absolute standardized difference
CTR: click-through rate
DID: difference in difference
KPI: key performance indicator
PSW: propensity score weighting
SNAP: Supplemental Nutrition Assistance Program
USDA: US Department of Agriculture
WIC: Special Supplemental Nutrition Program for Women, Infants, and Children
