# Supplementary material for: Using Social Media Marketing to Improve Retention of Children in the Special Supplemental Nutrition Program for Women, Infants, and Children: Implementation Study
Source: JMIR Public Health Surveill. 2025 Dec 10;11:e77172. doi: 10.2196/77172 (PMC12694944; doi:10.2196/77172)
Supplement: Multimedia Appendix 1 [file publichealth-v11-e77172-s001.docx]

Table S1**.** Absolute Standardized Differences in Characteristics (unweighted and weighted) in Miami-Dade WIC innovation and comparison clinics at T1 vs. T2, in the overall sample among infants and among children.

|  |  |  |  |  |  |  |
| --- | --- | --- | --- | --- | --- | --- |
|  | **Absolute Standardized Difference (ASD)** | | | | | |
|  | **Innovation T2 vs. Innovation T1** | | **Innovation T2 vs. Comparison T1** | | **Innovation T2 vs. Comparison T2** | |
|  | **unweighted** | **weighted** | **unweighted** | **weighted** | **unweighted** | **weighted** |
| Overall Sample |  |  |  |  |  |  |
| TWIN | 0.0049 | 0.0031 | 0.0447 | 0.0044 | 0.0469 | 0.0010 |
| RACE_AIAN | 0.0121 | 0.0021 | 0.0171 | 0.0169 | 0.0058 | 0.0054 |
| RACE_ASIAN | 0.0085 | 0.0080 | 0.0478 | 0.0039 | 0.0389 | 0.0082 |
| RACE_BLACK | 0.0360 | 0.0006 | 0.6400 | 0.0012 | 0.6336 | 0.0057 |
| RACE_NHPI | 0.0257 | 0.0257 | 0.0498 | 0.0012 | 0.0522 | 0.0000 |
| RACE_WHITE | 0.0125 | 0.0033 | 0.7172 | 0.0056 | 0.7087 | 0.0022 |
| HISPANIC | 0.0161 | 0.0288 | 0.7586 | 0.0081 | 0.7578 | 0.0027 |
| MULTIRACIAL | 0.0448 | 0.0003 | 0.0497 | 0.0067 | 0.0470 | 0.0054 |
| PRIM_LANG | 0.0802 | 0.0077 | 0.6872 | 0.0133 | 0.7123 | 0.0091 |
| TRANSLATION | 0.0132 | 0.0022 | 0.0172 | 0.0030 | 0.0045 | 0.0012 |
| TANF | 0.0244 | 0.0007 | 0.0316 | 0.0042 | 0.0067 | 0.0015 |
| SNAP | 0.2570 | 0.0115 | 0.1618 | 0.0131 | 0.4000 | 0.0255 |
| MEDICAID | 0.1013 | 0.0024 | 0.1234 | 0.0063 | 0.1866 | 0.0119 |
| INFANT | 0.0259 | 0.0532 | 0.0315 | 0.0252 | 0.0393 | 0.0826 |
| C1 | 0.0020 | 0.0215 | 0.0090 | 0.0088 | 0.0005 | 0.0331 |
| C2 | 0.0422 | 0.0211 | 0.0542 | 0.0161 | 0.0510 | 0.0335 |
| NUM_WIC | 0.0188 | 0.0034 | 0.0937 | 0.0097 | 0.0943 | 0.0035 |
| Mean ASD | 0.0427 | 0.0115 | 0.2079 | 0.0087 | 0.2227 | 0.0137 |
|  |  |  |  |  |  |  |
| Infants |  |  |  |  |  |  |
| TWIN | 0.0607 | 0.0042 | 0.0679 | 0.0009 | 0.1014 | 0.0025 |
| RACE_AIAN | 0.0141 | 0.0121 | 0.0075 | 0.0175 | 0.0097 | 0.0397 |
| RACE_ASIAN | 0.0446 | 0.0347 | 0.0608 | 0.0012 | 0.0294 | 0.0132 |
| RACE_BLACK | 0.0149 | 0.0726 | 0.6774 | 0.0086 | 0.6609 | 0.0170 |
| RACE_NHPI | 0.0313 | 0.0313 | 0.0366 | 0.0025 | 0.0336 | 0.0000 |
| RACE_WHITE | 0.0089 | 0.0274 | 0.7440 | 0.0088 | 0.7204 | 0.0033 |
| HISPANIC | 0.0283 | 0.0015 | 0.7608 | 0.0096 | 0.7428 | 0.0054 |
| MULTIRACIAL | 0.0239 | 0.0509 | 0.0205 | 0.0007 | 0.0210 | 0.0115 |
| PRIM_LANG | 0.0102 | 0.0590 | 0.6543 | 0.0171 | 0.6757 | 0.0126 |
| TRANSLATION | 0.0141 | 0.0028 | 0.0392 | 0.0019 | 0.0210 | 0.0001 |
| TANF | 0.0413 | 0.0126 | 0.0688 | 0.0084 | 0.0130 | 0.0422 |
| SNAP | 0.8232 | 0.0043 | 0.1893 | 0.0457 | 1.0055 | 0.0308 |
| MEDICAID | 0.2217 | 0.0188 | 0.1374 | 0.0105 | 0.2929 | 0.0029 |
| NUM_WIC | 0.0192 | 0.0071 | 0.0670 | 0.0087 | 0.0760 | 0.0207 |
| **Mean ASD** | **0.0969** | **0.0242** | **0.2523** | **0.0101** | **0.3145** | **0.0144** |

| **Children** |  |  |  |  |  |  |
| --- | --- | --- | --- | --- | --- | --- |
| TWIN | 0.0209 | 0.0018 | 0.0357 | 0.0060 | 0.0214 | 0.0030 |
| RACE_AIAN | 0.0251 | 0.0022 | 0.0291 | 0.0114 | 0.0126 | 0.0027 |
| RACE_ASIAN | 0.0078 | 0.0119 | 0.0401 | 0.0086 | 0.0438 | 0.0044 |
| RACE_BLACK | 0.0456 | 0.0056 | 0.6214 | 0.0050 | 0.6206 | 0.0023 |
| RACE_NHPI | 0.0223 | 0.0223 | 0.0563 | 0.0015 | 0.0609 | 0.0000 |
| RACE_WHITE | 0.0136 | 0.0010 | 0.7036 | 0.0032 | 0.7033 | 0.0027 |
| HISPANIC | 0.0092 | 0.0268 | 0.7573 | 0.0068 | 0.7659 | 0.0054 |
| MULTIRACIAL | 0.0547 | 0.0054 | 0.0640 | 0.0099 | 0.0596 | 0.0051 |
| PRIM_LANG | 0.1176 | 0.0071 | 0.7040 | 0.0101 | 0.7305 | 0.0131 |
| TRANSLATION | 0.0222 | 0.0031 | 0.0103 | 0.0036 | 0.0002 | 0.0021 |
| TANF | 0.0194 | 0.0000 | 0.0195 | 0.0023 | 0.0150 | 0.0069 |
| SNAP | 0.0174 | 0.0001 | 0.1426 | 0.0079 | 0.1674 | 0.0117 |
| MEDICAID | 0.0062 | 0.0062 | 0.1130 | 0.0037 | 0.1077 | 0.0046 |
| C1 | 0.0182 | 0.0036 | 0.0313 | 0.0032 | 0.0244 | 0.0037 |
| C2 | 0.0431 | 0.0025 | 0.0563 | 0.0081 | 0.0481 | 0.0021 |
| NUM_WIC | 0.0197 | 0.0016 | 0.1090 | 0.0092 | 0.1059 | 0.0037 |
| Mean ASD | 0.0289 | 0.0063 | 0.2183 | 0.0063 | 0.2180 | 0.0046 |

Abbreviations used: AIAN, American Indian or Alaskan Native; NHPI, Native Hawaiian or Pacific Islander; PRIM_LANG, primary language other than English; C1, child 13-23 months; C2, child 24-35 months; Num_WIC, number of children in household.
